# Synthesis, Bioevaluation and Molecular Dynamic Simulation Studies of Dexibuprofen–Antioxidant Mutual Prodrugs

**DOI:** 10.3390/ijms17122151

**Published:** 2016-12-21

**Authors:** Zaman Ashraf, Alamgeer, Raqiqatur Rasool, Mubashir Hassan, Haseeb Ahsan, Samina Afzal, Khurram Afzal, Hongsik Cho, Song Ja Kim

**Affiliations:** 1 Department of Chemistry, Allama Iqbal Open University, Islamabad 44000, Pakistan; mzchem@yahoo.com (Z.A.); raqiqa_chem@yahoo.com (R.R.); 2 Department of Pharmacology, Faculty of Pharmacy University of Sargodha, Sargodha 40100, Pakistan; alam_yuchi@yahoo.com (A.); haseebahsan110@yahoo.com (H.A.); 3 Department of Biology, College of Natural Sciences, Kongju National University, Gongju 314701, Korea; mubashirhassan_gcul@yahoo.com; 4 Faculty of Pharmacy, BahauddinZakria University, Multan 60800, Pakistan; samina.afzal@bzu.edu.pk (S.A.); khurram.afzal@bzu.edu.pk (K.A.); 5 Department of Orthopaedic Surgery & Biomedical Engineering, University of Tennessee Health Science Center-Campbell Clinic, Memphis, TN 38104, USA; hcho4@uthsc.edu; 6 Research 151, Veterans Affairs Medical Center, Memphis, TN 38104, USA

**Keywords:** nonsteroidal anti-inflammatory drugs (NSAIDs), mutual prodrugs, dexibuprofen, antioxidants, in vitro hydrolysis, pharmacological activity, molecular dynamics simulation

## Abstract

Dexibuprofen–antioxidant conjugates were synthesized with the aim to reduce its gastrointestinal effects. The esters analogs of dexibuprofen **5a**–**c** were obtained by reacting its –COOH group with chloroacetyl derivatives **3a**–**c**. The in vitro hydrolysis data confirmed that synthesized prodrugs **5a**–**c** were stable in stomach while undergo significant hydrolysis in 80% human plasma and thus release free dexibuprofen. The minimum reversion was observed at pH 1.2 suggesting that prodrugs are less irritating to stomach than dexibuprofen. The anti-inflammatory activity of **5c** (*p* < 0.001) is more significant than the parent dexibuprofen. The prodrug **5c** produced maximum inhibition (42.06%) of paw-edema against egg-albumin induced inflammation in mice. Anti-pyretic effects in mice indicated that prodrugs **5a** and **5b** showed significant inhibition of pyrexia (*p* < 0.001). The analgesic activity of **5a** is more pronounced compared to other synthesized prodrugs. The mean percent inhibition indicated that the prodrug **5a** was more active in decreasing the number of writhes induced by acetic acid than standard dexibuprofen. The ulcerogenic activity results assured that synthesized prodrugs produce less gastrointestinal adverse effects than dexibuprofen. The ex vivo antiplatelet aggregation activity results also confirmed that synthesized prodrugs are less irritant to gastrointestinal mucosa than the parent dexibuprofen. Molecular docking analysis showed that the prodrugs **5a**–**c** interacts with the residues present in active binding sites of target protein. The stability of drug–target complexes is verified by molecular dynamic simulation study. It exhibited that synthesized prodrugs formed stable complexes with the COX-2 protein thus support our wet lab results. It is therefore concluded that the synthesized prodrugs have promising pharmacological activities with reduced gastrointestinal adverse effects than the parent drug.

## 1. Introduction

Ibuprofen, (*R*,*S*-2-(4-isobutylphenyl)-propionic acid) a non-steroidal anti-inflammatory drug exists in two enantiomeric forms which are present in the racemate [1]. Dexibuprofen, *S*-(+)-ibuprofen is pharmacologically more active than racemic ibuprofen which has equal quantities of *R*-(−) and *S*-(+) enantiomers [2]. In the dose ratio of 1:0.5 (Racemic ibuprofen vs. dexibuprofen), at least equivalent efficacy was proven in acute mild to severe somatic and visceral pain models. The physico-chemical properties of dexibuprofen are also different than the racemic ibuprofen. Dexibuprofen has a slower dissolution rate in the simulated gastric and enteric juices compared with the racemic ibuprofen [3]. Dexibuprofen possesses comparable pharmacological efficacy and tolerability to that of diclofenac, naproxen and celecoxib [4].

The most commonly encountered adverse effects of nonsteroidal anti-inflammatory drugs (NSAIDs) including dexibuprofen are stomach ulceration, bleeding, perforation and gastrointestinal (GI) irritations [5,6]. The free carboxylic acid group (–COOH) present in dexibuprofen is responsible for the GI side effects [7,8]. Previously, a number of prodrugs of ibuprofen have been designed to temporarily mask the free carboxylic acid group to overcome gastrointestinal irritation [9,10,11] but so far no single NSAIDs available in market without gastrointestinal adverse effects. Esters and amides functionalities have been frequently employed for the preparation of prodrugs because they can easily be cleaved in to active drug by enzymatic hydrolysis [12,13]. Mutual prodrug consists of two pharmacologically active agents coupled together in such a way to get synergistic action.

A number of prodrugs of ibuprofen have been reported [14,15,16] but very few mutual prodrugs of dexibuprofen were reported [17,18]. The current study was undertaken to synthesize novel mutual prodrugs of dexibuprofen to reduce its gastrointestinal effects while maintaining the pharmacological profile. The carboxylic group of dexiboprofen was temporarily masked by synthesizing its ester analogs with naturally occurring phenolic antioxidants sesamol and umbelliferon as well as alcoholic compound menthol. The selected promoieties have traditional medicinal uses and flavoring properties with well-documented safety profiles [19]. The in vitro hydrolysis of the synthesized mutual prodrugs (**5a**–**c**) was carried out in aqueous buffers and in 80% human plasma pH 7.4 for ensuring their stability. Pharmacological screenings like anti-inflammatory, analgesic, antipyretic and ulcerogenic activities of these prodrugs were evaluated and results were compared with standard dexibuprofen. The ex vivo platelet aggregation activity of the synthesized prodrugs was also carried out. Molecular docking and simulation studies were performed to predict the binding affinity of synthesized prodrugs with COX-2.

## 2. Result and Discussion

### 2.1. Synthesis of Prodrugs

**Scheme 1:**
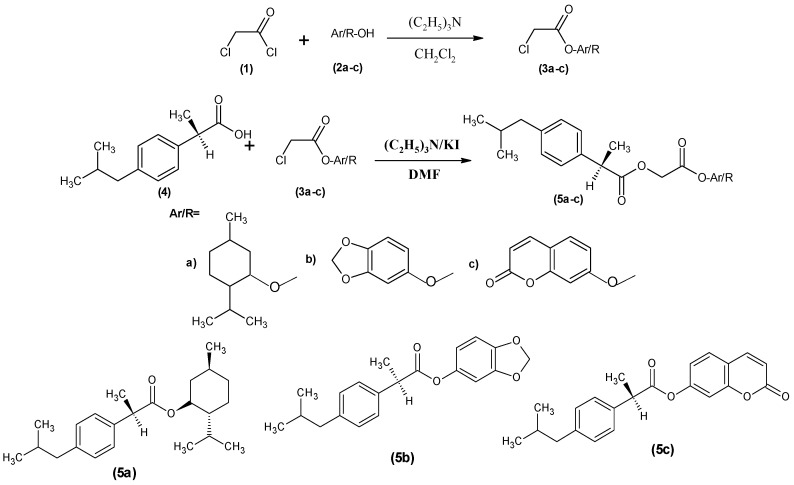
Synthesis and chemical structures of dexibuprofen–antioxidant mutual prodrugs (**5a**–**c**).

The mutual prodrugs synthesis is beneficial as it minimizes the undesirable effects of a drug along with increase the pharmacological response. Dexibuprofen–antioxidants mutual prodrugs have been synthesized by following the previously reported method with slight modification [20]. For the first time natural antioxidants menthol, sesamol and umbelliferon were used for the preparation of mutual prodrugs of dexibuprofen. Chloroacetyl chloride was condensed with these natural antioxidants **2a**–**c** to afford their chloroacetyl chloride derivatives **3a**–**c**. Dexibuprofen was then reacted with derivatives **3a**–**c** to afford mutual prodrugs **5a**–**c** (Scheme 1). The structures of the synthesized prodrugs were confirmed by FTIR, ^1^H-NMR, ^13^C-NMR and Mass spectral data. The FTIR spectral data showed the characteristic absorption for C=O at 1730–1718 cm^−1^ with disappearance of –OH for carboxylic group which confirmed the formation of ester prodrugs.

### 2.2. Hydrolysis Studies of Mutual Prodrugs Dexibuprofen

**Figure 1:**
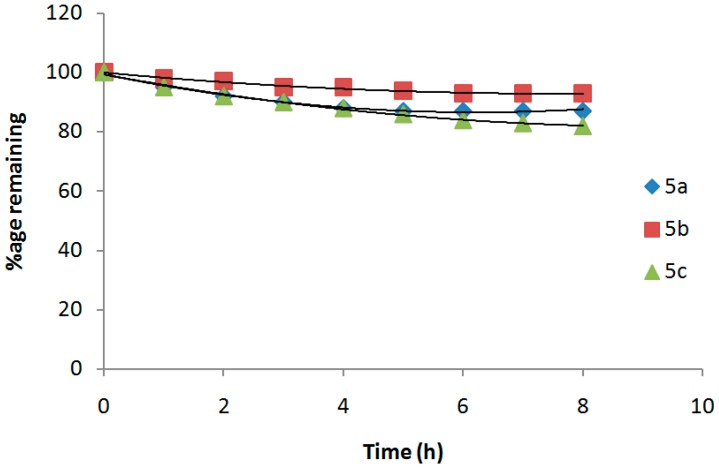
In vitro hydrolytic pattern of the ester prodrugs (**5a**–**c**) in simulated intestinal fluid (SIF) (pH 7.4).

**Figure 2:**
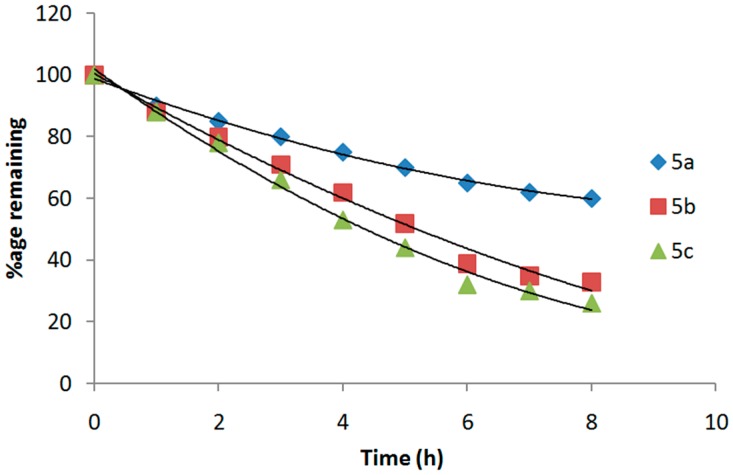
In vitro hydrolytic pattern of the ester prodrugs (**5a**–**c**) in 80% human plasma (pH 7.4).

The in vitrohydrolysis of the synthesized prodrugs to free dexibuprofen was performed at pH 1.2 and 7.4 to mimic the stomach and intestinal environment respectively. The release of free drug by hydrolysis of the prodrugs was also studied in 80% human plasma at pH 7.4 to mimic the pH of blood. The prodrugs at gastric pH (SGF, pH 1.2) are not hydrolyzed to free dexibuprofen while at higher (SIF pH 7.4) hydrolysis of the prodrugs is higher resulting in increase the amount free dexibuprofen. The dexibuprofen produced on hydrolysis after 1 h (in SIF, pH 7.4) of prodrugs **5a**–**c** was found from 6.34% to 18.02%. The amount of dexibuprofen regenerated on hydrolysis after 1 h (in 80% human plasma, pH 7.4) was found to be 69.4%, 61.5% and 64.7% in prodrugs **5a**–**c**, respectively. All of the mutual prodrugs showed very encouraging hydrolysis rate in 80% human plasma (pH 7.4) and the regeneration of active drug was found to be 61% to 92%. The In vitro hydrolytic patterns of the ester prodrugs **5a**–**c** in SIF (pH 7.4) and in 80% human plasma (pH 7.4) are presented in Figure 1 and Figure 2, respectively.

### 2.3. Pharmacology

#### Anti-Inflammatory Activity

Anti-inflammatory activity results confirmed that there was a gradual increase in paw volume of mice in the control group. However, in the treated groups, all prodrugs **5a**–**c** produced a significant reduction in edema formation. The prodrug **5c** exhibited anti-inflammatory effects by significantly (*p* < 0.001) inhibiting the paw-edema at 4 h of post carrageenan induced inflammation with percentage inhibition of (28.99%). However, prodrugs **5a** and **5b** produced comparatively less anti-inflammatory response (*p* < 0.01) than standard dexibuprofen (Table 1 and Table 2). Carrageenan induce edema in bi-phase, the duration of first phase is 0–2.5 h post injection of agent. During this phase, different inflammatory mediators such as histamine, serotonin and bradykinin are released that cause vasodilation [21], whereas during the second phase inflammatory mediators such as prostaglandins are released. The experimental results showed that all synthetic prodrugs exhibited very significant inhibitory effect (*p* < 0.001) in second phase of induction of edema. Thus, this effect could be due to inhibitory effect on cyclooxygenase enzyme that leads to decreased production of Prostaglandins. The anti-inflammatory effect of standard dexibuprofen is also due to inhibition of both COX-1 and COX-2 [22].

Prodrugs **5a**–**c** in egg induced inflammation also showed significant (*p* < 0.001) anti-inflammatory effects at 3 h post albumin induced inflammation with maximum inhibition (42.06%) produced by **5c**. The standard drug dexibuprofen also produced significant (*p* < 0.001) anti-inflammatory effects at 3 h after the administration of egg albumin. Anti-inflammatory effects exhibited by prodrugs were comparable to parent dexibuprofen. Previously, it has been reported that injection of egg albumin caused release of histamine and 5-HT which lead to inflammation. Histamine is a vasodilator and causes increase in vascular permeability [23,24]. The anti-inflammatory effects of synthesized prodrugs might be due to inhibiting the release of mediators such as histamine which cause swellings associated with inflammation [25].

### 2.4. Analgesic Activity

Dexibuprofen mutual prodrugs **5a**–**c** exhibited a significant (*p* < 0.001) reduction in writhings induced by acetic acid. The mean percent inhibition indicated that the prodrug **5a** was more active in decreasing the number of writhes induced by acetic acid than standard dexibuprofen. On the other hand, **5b** and **5c** showed comparable analgesic activity to dexibuprofen. On the contrary, all prodrugs displayed higher analgesic activity than standard dexibuprofen in formalin induced licking in mice. The analgesic response of synthetic prodrugs in formalin induced licking in mice was in order of **5a** > **5b** > **5c** (Table 3).

In acetic acid induced writhing model, abdominal constriction response is due to peritoneal receptors [26]. It is reported that level of prostaglandin E2 (PGE2) and PGF2 in peritoneal fluids has been increased [27] as well as lipoxygenase products [28] when acetic acid is administered to induce writhing. The analgesic activity of prodrugs might be due to inhibition of synthesis and release of PGs and other endogenous substances as already proposed. Likewise, formalin produces response in two phases; the first phase is due to stimulation of nociceptors while second phase is of inflammatory pain origin [29]. The mutual prodrugs at a dose of 20 mg/kg significantly (*p* < 0.001) reduced licking time in mice. The analgesic activity of prodrugs **5a**–**c** was evaluated on the first and second phase of formalin-induced paw licking test. The results verified that these analgesic effects may be due to its central action as proposed previously by Ghannadi et al., 2005 [30].

### 2.5. Antipyretic Activity

Anti-pyretic effects in mice indicated that prodrugs **5a** and **5b** showed significant inhibition of pyrexia (*p* < 0.001). Prodrug **5c** showed significant inhibition of pyrexia after 2 h induction of pyrexia while standard dexibuprofen showed less significant (*p* < 0.05) inhibition of pyrexia. It has been well established that yeast induces pyrexia by increasing the production of prostaglandins (PGE_2_), which raises the set point of thermoregulatory center in hypothalamus [31]. Therefore, it is proposed that **5b** may likely to reduce pyrexia by decreasing prostaglandin E2 concentration in brain particularly in the hypothalamus [32] or by increasing the production of the body′s own antipyretic substances such as vasopressin and arginine [33]. Antipyretic effects of dexibuprofen may be due to the action on the hypothalamus, resulting in an increased peripheral blood flow, vasodilation and subsequent heat dissipation. Table 4 depicts the antipyretic activity results of the synthesized mutual prodrugs **5a**–**c**.

### 2.6. Ulcerogenic Activity

To find adverse effects on gastrointestinal mucosa the ulcerogenic activity of the synthesized dexibuprofen–antioxidant mutual prodrugs **5a**–**c** was performed. The ulcer index (UI) of animals administered with dexibuprofen prodrugs **5a**–**c** were compared with that of dexibuprofen and results were evaluated using one way ANOVA (followed by Tukey) test (Table 5). Ulcer index shown by dexibuprofen treated animals is 2.89 and is due to direct contact mechanism as well as prostaglandin inhibition. All of the synthesized dexibuprofen–antioxidant mutual prodrugs **5a**–**c** significantly reduce the gastrotxicity of dexibuprofen as the ulcer index produced by synthesized prodrugs is less than parent dexibuprofen. The present study did not include the side effects associated with the long term use of the prodrugs.

### 2.7. Ex Vivo Antiplatelet Aggregation Activity

**Figure 3:**
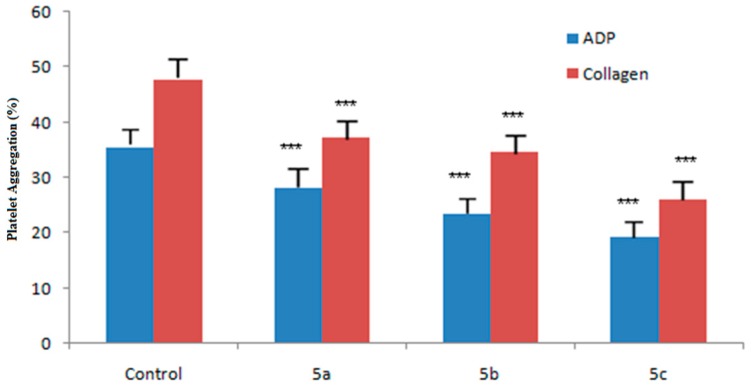
Antiplatelet aggregation activity of synthesized prodrugs (**5a**–**c**) in rats administered orally. The platelet rich plasma was obtained from the blood 90 min after prodrugs (100 mg/kg) were orally administered into Sprague-Dawley (SD) rats, and platelet aggregation was induced by adenosinediphosphate (ADP) (1.3 µmol/L) or collagen (32.8 µg/mL), Significant at *** = *p* < 0.001.

Dexibuprofen–antioxidant mutual prodrugs (**5a**–**c**) significantly inhibited platelet aggregation of rat platelet rich plasma induced by ADP or collagen. Figure 3 displays the inhibitory effects of synthesized prodrugs **5a**–**c**. All of the synthesized prodrugs **5a**–**c** having higher potential to inhibit the platelet aggregation than the positive control ASA. The prodrug **5c** exhibited most potent inhibitory effects compared to other synthesized prodrugs.

### 2.8. Molecular Docking Analysis

Molecular docking of the parent dexibuprofen and synthesized prodrugs **5a**–**c** against target protein have been performed. From these data, we can find the role of attached promoities in the receptor interactions. This is helpful to design new, more potent derivatives of dexibuprofen.

#### Cyclooxygenase-2 Evaluation 

COX-2 is a multi-domain protein (A, B, C and D), comprising 552 residues. The crystal structure of COX-2 consists of 44% helices (248 residues), 11% β sheet (64 residues) and 43% coil (240 residues). The X-ray diffraction study confirmed its resolution 2.19 Å, R-value 0.255 and unit cell crystal dimensions like length and angles of coordinates. The unit cell length values and angle dimensions were observed for a = 180.27 Å, b = 134.27 Å and c = 122.61 Å with α, β and γ angles 90°, 90° and 90° respectively. The reliability and efficacy of cox-2 3D structure was confirmed by Ramachandran plots. The Ramachandran plots indicated that the 97.3% of all residues were present in favored regions and 100.0% residues were in allowed regions (Figure S1).

### 2.9. Prodrug Ligands Evaluation

The prodrugs **5a**–**c** were analyzed computationally to predict their basic molecular and chemo-informatic properties (Table S1). Literature study established a standard value for molar refractivity (MR) (40 to 130 cm^3^), molecular weight (160 to 480 g/mol) and PSA (<89 Å^2^) [34,35]. The **5a**–**c** results showed that MR (116.26, 102.69 and 110.04 cm^3^) and PSA (40.80, 58.27, and 61.56 Å^2^) predicted values are much effective compared to standard values. Lipinski′s rule of five (RO5) evaluations also presented their good therapeutic potential of ligands. It has been studied that that poor permeation is observed in the presence of greater values (>10, <5) of HBA and HBD, respectively [36]. The computational analysis showed that all prodrugs **5a**–**c** have<10 HBA and <5 HBD and comparable values for molecular weight (g/mol) and log*P* with standard values (<500 g/mol and <5). The RO5 also rationalizes that molecules having poor absorption are the results of >5HBD, >10HBA, MWT over 500, and log*P* more than 5. However, there are few existing drugs that violate the RO5 rule and have therapeutic potential [37,38]. **5b** showed good drug likeness value (1.22) as compared to **5a** (0.83) and (**5b**) (1.04). Overall, these predicted values justify the significance of **5a**–**c** compounds as potential drug candidates.

### 2.10. Binding Energy Analysis

The prodrugs docked complexes of dexibuprofen and **5a**–**c** were analyzed and evaluated on minimum energy (kcal/mol) value as mentioned in Table 6. Among all the docked complexes the **5b** showed lowest binding energy value (−9.90 kcal/mol) as compared to others. Moreover, **5a** and **5c** also possess good binding energy values (−8.90 and −9.40 kcal/mol, respectively). The binding energy value of the parent dexibuprofen is −8.60 kcal/mol. Based upon the docking energy values it is proposed that all of synthesized prodrugs have comparable therapeutic potential. The binding energy values of all the docked complexes are also shown in the Table S2.

### 2.11. Hydrogen Bonding Analysis

**Figure 4:**
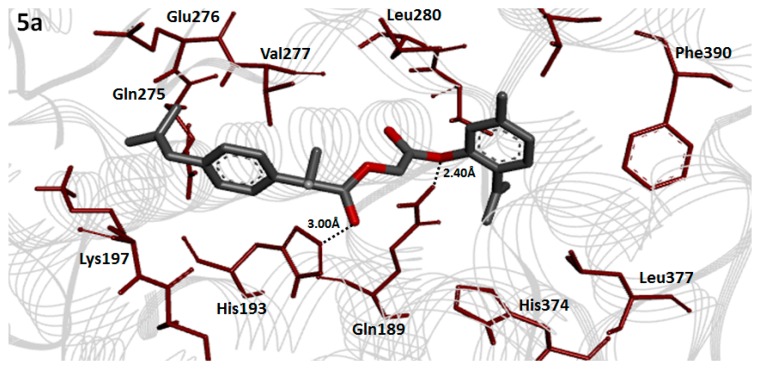
Docking interactions of (**5a**) with receptor. The COX-2 protein chain is highlighted in light grey color in wire format. The dark grey color showed the ligand structure while the embedded red colors showed oxygen moiety. The binding pocket interacted residues are highlighted in dark red color and black dotted lines justify the hydrogen bondings. Two hydrogen bonds were observed with GLN189 and HIS193 at a distance 2.40 and 3.00 Å, respectively.

**Figure 5:**
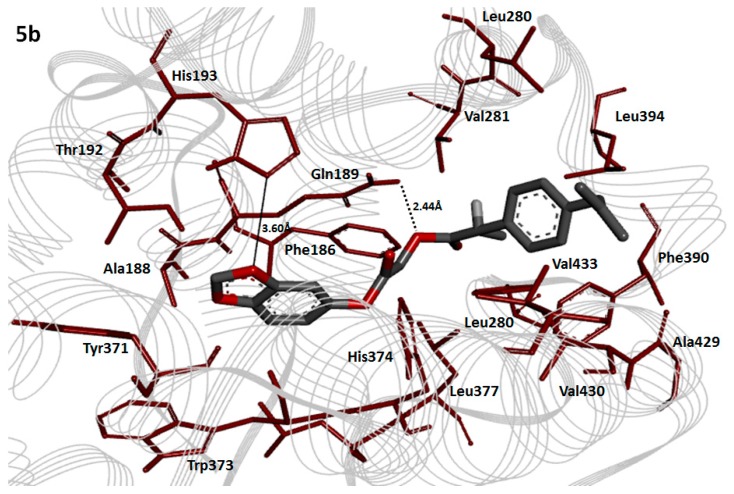
Docking interactions of (**5b**) with receptor. The COX-2 protein chain is highlighted in light grey color in wire format. The dark grey color showed the ligand structure while the embedded red colors showed oxygen moiety. The binding pocket interacted residues are highlighted in dark red color and black dotted lines justify the hydrogen bondings. Two hydrogen bonds were observed with GLN189 and HIS193 at a distance 2.44 and 3.60 Å, respectively.

**Figure 6:**
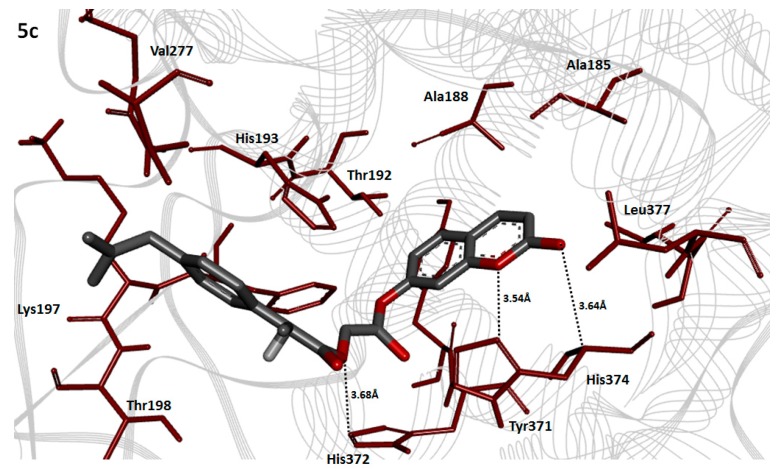
Docking interactions of (**5c**) with receptor. The COX-2 protein chain is highlighted in light grey color in wire format. The dark grey color showed the ligand structure while the embedded red colors showed oxygen moiety. The binding pocket interacted residues are highlighted in dark red color and black dotted lines justify the hydrogen bondings. Two hydrogen binds were observed with HIS374 at a distances of 3.64 and 3.54 Å and one with HIS372 at a distance 3.68 Å, respectively.

**Figure 7:**
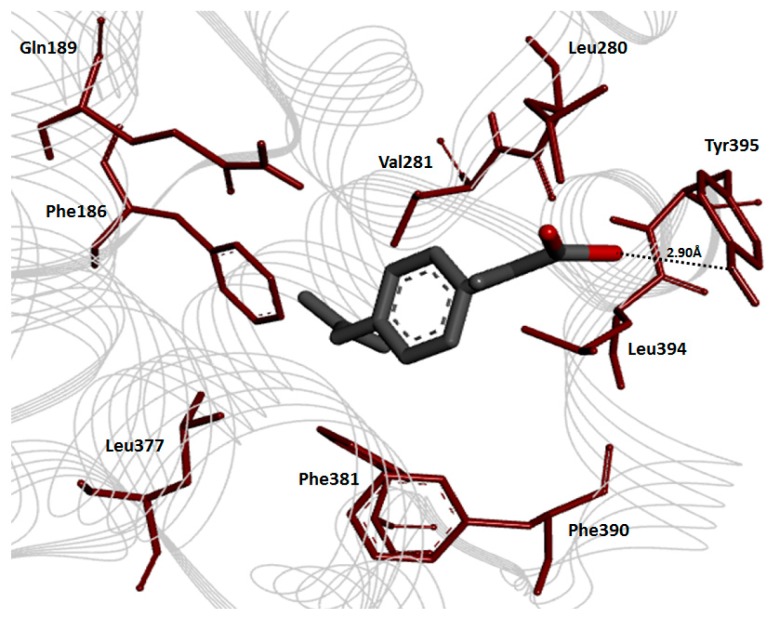
Docking interactions of dexibuprofen with receptor. The COX-2 protein chain is highlighted in light grey color in wire format. The dark grey color showed the dexibuprofen structure while the embedded red colors showed oxygen moiety. The binding pocket interacted residues are highlighted in dark red color and black dotted lines justify the hydrogen bondings.

The hydrogen bonding analyses showed that dexibuprofen and synthesized prodrugs **5a**–**c** directly interact with the binding pocket residues of targeted protein. Figure 4 and Figure 5 showed that compound **5a** and **5b** interact with same residues GLN189 and HIS193 in the active binding site of targeted protein. The ester carbonyl oxygen and phenoxy oxygen in **5a** formed hydrogen bonds with GLN172 and HIS176 at a bonding distances 2.40 and 3.00 Å respectively. Similarly, the ester and methylene oxygen in **5b** interact through hydrogen bonds with same residues GLN189 and HIS193 at bonding distances 2.44 and 3.60 Å, respectively. The comparative analysis showed that both produgs **5a** and **5b** confined at the same position in the active region of target proteins. The binding energy values (−8.90 and −9.90 kcal/mol) and strong binding interactions assure their greater affinity to bind at active binding site of COX-2. The 5c-receptor docked complex showed the conformational state of prodrug molecule with hydrogen bonds interactions near the receptor binding pocket (Figure 6). The (**5c**) docking result showed that three hydrogen bonds were observed at HIS374 and HIS372 residues position in the target protein. The coumarin lactonic oxygens forms two hydrogen bonds with HIS374 having bond length 3.64 and 3.54 Å. Similarly, the ester oxygen of same compound also forms hydrogen bond with HIS355 with bond distance 3.68 Å. The dexibuprofen docked complex with target protein is presented in Figure 7 which showed that the dexibuprofen and synthesized prodrugs interacts in the active binding site residues. The carboxylic oxygen in dexibuprofen interacted with TYR295 at a bonding distance of 2.90 Å. The literature study also showed that binding pocket residues are common in our present result, which justified the significance of ligands binding [39].

### 2.12. Molecular Dynamics (MD) Simulations Analysis

**Figure 8:**
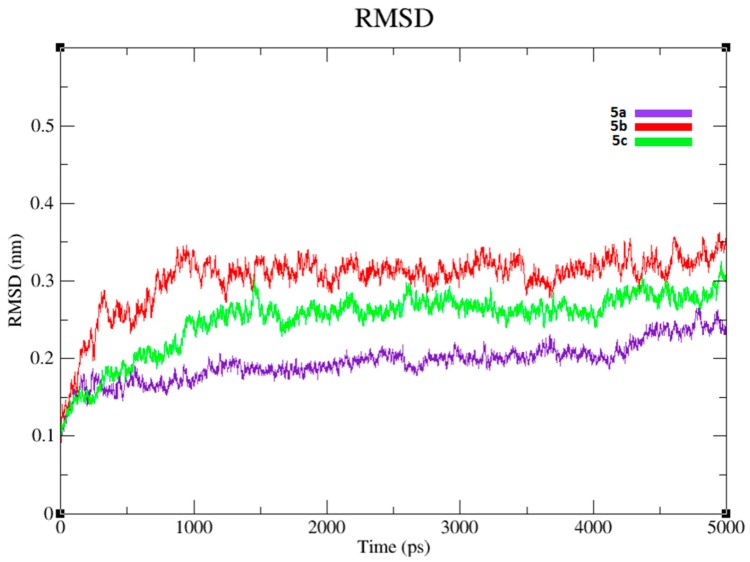
Root Mean Square Deviation (RMSD) graphs of (**5a**–**c**) complexes at different time scale. The backbones with indigo, red and green represent (**5a**–**c**) complexes, respectively.

To evaluate the residual flexibility and structural behavior of target protein, we incorporated **5a**–**c** docking complexes in Gromacs 4.5.4 and performed MD at 5 ns. The Root Mean Square Deviation (RMSD) played a significant role in protein stability [40]. The generated RMSD graphs of all three complexes **5a**–**c** depicted the backbone flexibility of the targeted protein (Figure 8). Furing the over all simulation period, RMSD increasing trend was observed in all three complexes from 0 to 1000 ps. In **5a** simulation, the RMSD increased and stabilized at 1000 ps. It remained stable in the simulation period from 1000 to 4000 ps, but showed a little increasing trend at 5000 ps. In case of **5b** complex, the RMSD graph is increased gradually from 0 to 1000 ps and then gains stability throughout the simulation period. However, prodrug **5c** RMSD graph showed an increasing trend from 0 to 1500 ps and then remains stable in the simulation period with little fluctuations. From the RMSD analysis, the **5b** structure seemed more stable compared to the **5a** and **5c** structures due to lower fluctuation of RMSD from 1000 to 5000 ps. The predicted results showed that attachment of prodrugs at the active site of the protein does not disturb the protein backbone stability. The dexibuprofen RMSD value was calculated by match maker command in Chimera tool. The dexibuprofen docked structure was superimposed against the crystal structure. The predicted RMSD value (0–1 Å) of the docked dexibuprofen structure showed the efficient binding within the active binding site. It has been reported that the higher RMSD value showed more deviation from the template structure [41].

**Figure 9:**
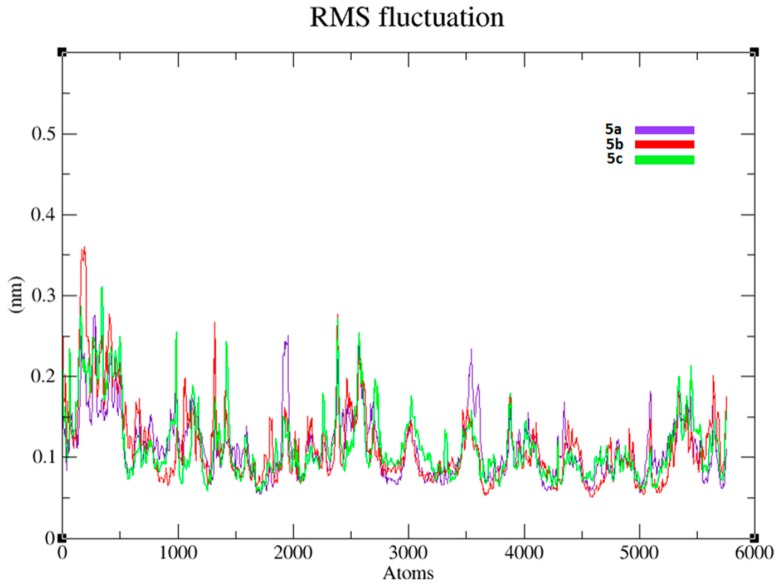
Root Mean Square Fluctuations (RMSF) graph of (**5a**–**c**) complexes at different time scale. The backbones with indigo, red and green represent the (**5a**–**c**) complexes, respectively.

**Figure 10:**
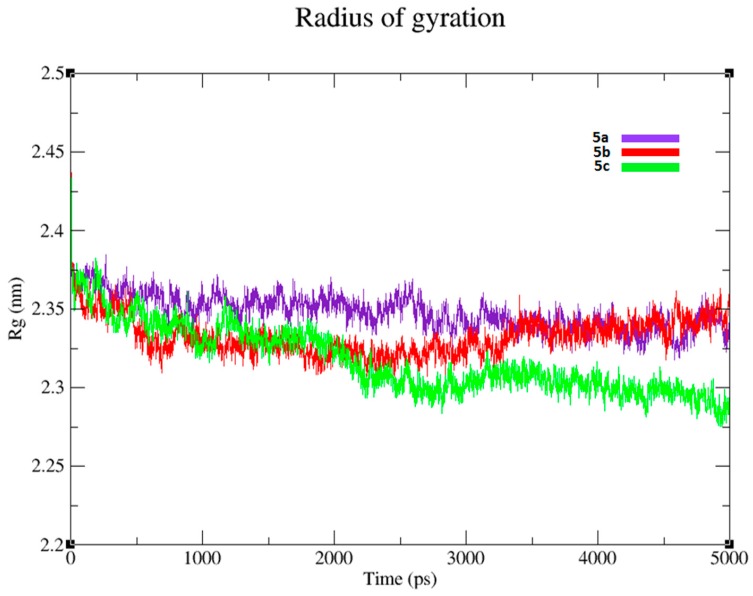
Radius of gyration (*R*_g_) graph of prodrugs (**5a**–**c**).

The residual fluctuations in the **5a**–**c** docking complexes were observed by Root Mean Square Fluctuation (RMSF). The predicted RMSF graphs of C-α atom of all three complexes were plotted against the interacted residues based on the trajectory period of MD simulation. All the residues in docking complexes were fluctuated around 0.1–0.3 nm in the simulation period. All of the prodrugs showed little changes in residual fluctuation at different loop regions. The RMSF results also reflect that N-terminal lobe of COX-2 is exhibited more fluctuations than C-terminus (Figure 9). The compactness of protein was observed by radius of gyration (*R*_g_). The predicted results of COX-2 justified that *R*_g_ value is little fluctuated between 2.3 and 2.4 nm throughout the simulation time 0–5000 ps. The comparative results showed that residual backbone and folding of COX-2 was steadily stable after binding the inhibitor **5b** compared to others (Figure 10).

## 3. Material and Methods

### 3.1. Chemicals

Formalin (MERCK, Darmstadt, Germany), Carrageenan (Sigma Chemical Company. St. Louis, MO, USA), Dried yeast (Sigma Chemical Company), and Normal saline (0.9% NaCl) were used.

### 3.2. Experimental

The digital Gallenkamp (SANYO, Calgary, AB, Canada) melting point apparatus model MPD BM 3.5 was used to determine the melting points. The FTS 3000MX and Bruker AM-300 spectrophotometers were used for FTIR and NMR spectral analysis. The ^1^H-NMR and ^13^C-NMR spectral analysis were performed as CDCl_3_ solutions. The Agilent 6460 Series Triple Quadrupole instrument (Agilent, Santa Clara, CA, USA) was used to determine the molecular masses of the synthesized prodrugs. The electrospray ionization technique in the positive ion mode (ESI+) was employed for ionization. The purity of the final products was ascertained by preparative thin layer chromatography (TLC) on silica gel HF-254.

### 3.3. Synthesis of Antioxidant Chloroacetyl Derivatives (**3a**–**c**)

#### General Procedure

Antioxidants (0.01 mol) and triethylamine (0.01 mol, 1.0 mL) were dissolved in anhydrous dichloromethane (25 mL) at 0 to −5 °C. To this solution, chloroacetyl chloride (0.01 mol, 0.0796 mL) in dry dichloromethane was added drop wise over a period of 1 h maintaining the temperature constant. The reaction mixture was stirred for 5 h at room temperature. After the completion of reaction the products were extracted with ethyl acetate and washed with 5% HCl, 5% sodium hydroxide and with saturated NaCl solution. The ethyl acetate layer was rotary evaporated to afford the antioxidant chloroacetyl derivatives. All of the antioxidant chloroacetyl derivatives **3a**–**c** were synthesized starting with natural antioxidants **2a**–**c** by employing this general procedure. The synthesized compounds **3a**–**c** were purified by recrystallization in petroleum ether and ethyl acetate mixture (2:1).

*Synthesis of 2-isopropyl-5-methylcyclohexyl chloromethanoate* **3a**. Yield 71%, solid, m.p. 38–40 °C, *R*_f_ 0.55 (pet. ether:ethyl acetate 3:1), IR ν_max_ cm^−1^ (KBr): 2961 (C–H), 1739 (C=O ester), 1511 (C=C), 1196 (C–O), 788 (C–Cl).

*Synthesis of 3,4-(methylenedioxy)phenyl chloromethanoate* **3b**. Yield 82%, solid, m.p. 46–48 °C, *R*_f_ 0.6 (pet. ether:ethyl acetate 3:1), IR ν_max_ cm^−1^ (KBr): 2941 (C–H), 1767 (C=O ester), 1510 (C=C), 1165 (C–O), 837 (C–Cl).

*Synthesis of 2-oxo-2H-chromen-7-ylchloroethanoate* **3c**. Yield 81%, solid, m.p. 36–38 °C, *R*_f_ 0.59 (pet. ether:ethyl acetate 3:1), IR ν_max_ cm^−1^ (KBr): 2945 (C–H), 1767 (C=O ester), 1511 (C=C), 1154 (C–O), 788 (C–Cl).

### 3.4. Synthesis of Dexibuprofen Antioxidant Mutual Prodrugs **5a**–**c**

#### General Procedure

Antioxidant chloroacetyl derivatives (0.01 mol) and dexibuprofen (**4**; 2.06 g, 0.01 mol) were dissolved in dimethylformamide DMF (25 mL). Triethyl amine (0.01 mol, 1.0 mL) and potassium iodide (0.01 mol, 1.66 g) were added to the reaction mixture and stirred it overnight at room temperature. After the completion of reaction the mixture was poured into ice cold water and extracted with chloroform (4 × 25 mL). The combined organic layer was washed with 2% sodium thiosulfate, 5% HCl, 5% sodium hydroxide and finally with brine solution. The organic layer was dried over anhydrous sodium sulfate, filtered and the solvent was removed under reduced pressure to obtain the corresponding mutual prodrugs. The title mutual prodrugs **5a**–**c** were synthesized by following the above-mentioned general procedure starting with different antioxidants chloroacetyl derivatives **3a**–**c** (Scheme 1). The products were obtained as semisolids and were purified by preparative thin layer chromatography using petroleum ether and ethyl acetate as eluents (3:1).

*Synthesis of (S)-((2-isopropyl-5-methyl-5-methylcyclohexyl) carbonyl) methyl 2-(4-isobutylphenyl) propanoata* **5a**. Yield 76.5%, semisolid, *R*_f_ 0.64 (pet. ether:ethyl acetate 3:1), IR ν_max_ cm^−1^ (KBr): 2953 (C–H), 1745 (C=O ester), 1138 (C–O, ester); ^1^H-NMR (CDCl_3_, δ ppm): 1.01 (d, *J* = 7.4 Hz, 6H, H-7), 1.45 (q, *J* = 7.8 Hz, 1H, H-6′′), 1.50 (q, *J* = 4.1 Hz, 2H, H-5′′), 1.52 (q, *J* = 4.0 Hz, 2H, H-4′′), 1.55 (d, *J* = 3.3 Hz, 6H, H-1), 1.72 (m, 1H, H-3′′), 1.76 (t, *J* = 5.1 Hz, 2H ,H-2′′), 1.78 (m, 1H, H-2), 1.82 (septet, 1H, H-8), 2.25 (s, 3H, H-9), 2.43 (d, *J* = 7.2 Hz, 3H, H-4), 2.88 (d, *J* = 4.5 Hz, 2H, H-3), 2.95 (q, *J* = 7.8 Hz, 1H, H-1′′), 3.24 (s, 2H, H-6), 4.55 (q, *J* = 9.8 Hz, 1H, H-5), 7.06 (d, *J* = 8.1 Hz, 2H, H-2′), 7.24 (d, *J* = 7.2 Hz, 2H, H-1′); ^13^C-NMR (CDCl_3_, δ ppm): 26.15 (C-1), 31.65 (C-2), 44.63 (C-3), 25.89 (C-4), 40.63 (C-5), 167.38 (C-6), 50.18 (C-7),174.04 (C-8), 25.89 (C-9), 30.13 (C-10), 23.41 (C-11), 140.05 (C-1′), 127.23 (C-2′), 129.35 (C-3′), 137.17 (C-4′), 61.11 (C-1′′), 34.15 (C-2′′), 31.43 (C-3′′), 34.58 (C-4′′), 31.37 (C-5′′), 46.83 (C-6′′); ESI-MS: *m*/*z* 425 (43%) (M + 23) (M + Na), 247 (23%), 189 (100%), 161 (62%), 155 (43%), 133 (31%).

*Synthesis of (S)-((benzo(d)-(1,3)dioxol-6-yloxy) carbonyl) methyl-2-(4-isobutylphenyl) propanoate* **5b**. Yield 72.9%, semisolid, *R*_f_ 0.70 (pet. ether:ethyl acetate 3:1), IR ν_max_ cm^−1^ (KBr): 2957 (C–H), 1735 (C=O ester), 1147 (C–O, ester), 1105 (C–O ether); ^1^H-NMR (CDCl_3_, δ ppm): 1.05 (d, *J* = 6.1 Hz, 6H, H-1), 2.11 (m, 1H, H-2), 2.20 (d, *J* = 6.6 Hz, 3H, H-4), 2.67 (d, *J* = 7.2 Hz, 2H, H-3), 3.78 (q, *J* = 9.8 Hz, 1H, H-5), 4.89 (s, 2H, H-6), 5.80 (s, 2H, H-7), 6.40 (s, 1H, H-3′′), 6.53 (d, *J* = 7.7 Hz, 1H, H-1′′), 6.72 (d, *J* = 8.1 Hz, 1H, H-2′′), 7.15 (d, *J* = 7.8 Hz, 2H, H-2′, H-3′), 7.24 (d, *J* = 8.1 Hz, 2H, H-1′, H-4′); ^13^C-NMR (CDCl_3_, δ ppm): 20.81 (C-1), 23.43 (C-2), 44.62 (C-3), 18.24 (C-4), 43.25 (C-5), 61.90 (C-6), 157.64 (C-7), 160.90 (C-8), 99.82 (C-9), 137.21 (C-1′), 128.00 (C-2′, C-6′), 128.35 (C-3′, C-5′), 131.01 (C-4′), 143.70 (C-1′′), 112.29 (C-2′′), 113.74 (C-3′′), 144.81 (C-4′′), 148.92 (C-5′′), 105.43 (C-6′′); ESI-MS: *m*/*z* 407 (39%) (M + 23) (M + Na), 247 (25%), 189 (100%), 161 (54%), 137 (37%), 133 (26%).

*Synthesis of (S)-((2-oxo-2H-chromen-7-yloxy) carbonyl) methyl 2-(4-isobutylphenyl) propanoate* **5c**. Yield 68%, semisolid, *R*_f_ 0.75 (pet. ether:ethyl acetate 3:1), IR ν_max_ cm^−1^ (KBr): 2957 (C–H), 1730 (C=O ester), 1142 (C–O, ester); ^1^H-NMR (CDCl_3_, δ ppm): 1.49 (d, *J* = 6.5 Hz, 3H, H-4), 1.53 (d, *J* = 5.8 Hz, 6H, H-1), 2.22 (m, 1H, H-2), 2.66 (d, *J* = 6.8 Hz, 2H, H-3), 4.25 (q, *J* = 9.3 Hz, 1H, H-5), 5.13 (s, 2H, H-6), 6.15 (d, *J* = 6.1 Hz, 1H, H-4′′), 6.80 (d, *J* = 7.2 Hz, 1H, H-1′′), 6.83 (s, 1H, H-5′′), 7.24 (d, *J* = 7.0 Hz, 1H, H-2′′), 7.38 (d, *J* = 6.1 Hz, 1H, H-3′′), 7.39 (d, *J* = 8.7 Hz, 2H, H-2′, H-6′), 7.44 (d, *J* = 8.2 Hz, 2H, H-1′, H-4′); ^13^C-NMR (CDCl_3_, δ ppm): 20.81 (C-1), 25.34 (C-2), 49.62 (C-3), 16.50 (C-4), 40.21 (C-5), 173.15 (C-6), 69.29 (C-7), 174.44 (C-8), 137.73 (C-1′), 128.12 (C-2′), 129.39 (C-3′), 131.42 (C-4′), 150.01 (C-1′′), 116.34 (C-2′′), 125.21 (C-3′′), 117.08 (C-4′′), 140.64 (C-5′′), 109.50 (C-6′′), 160.92 (C-7′′), 147.64 (C-8′′), 108.28 (C-9′′); ESI-MS: *m*/*z* 431 (36%) (M + 23) (M + Na), 247 (30%), 189 (100%), 161 (63%), 134 (49%), 133 (32%).

### 3.5. In Vitro Hydrolysis

The in vitrohydrolytic studies of mutual prodrugs **5a**–**c** were performed in simulated fluid (pH 1.2 and 7.4) and in 80% human plasma (pH 7.4). These simulated fluids i.e., simulated gastric fluid (SGF pH 1.2) and simulated intestinal fluid (SIF pH 7.4) are extensively utilized in pharmaceutical industries for dissolution tests. Hydrochloric acid solution and potassium phosphate monobasic were used to prepare these media. The simulated gastric fluid is prepared by mixing the 0.2 M aqueous potassium chloride with a 0.2 M hydrochloric acid solution. The SGF was degassed after adjusting the final pH. The monobasic potassium phosphate buffer was prepared in water and final pH 7.4 was adjusted by slow addition of potassium hydroxide solution. To prepare the stock solution, 10 mg of each prodrug was mixed in 90 mL of SGF (pH 1.2) or SIF (pH 7.4). The 15 mL of this stock solution was taken repeatedly and kept in test tubes maintained at 37 ± 0.5 °C. From these test tubes some solution was taken after a fixed time interval (0.5, 1, 2 up to 8 h) and was transferred to micro centrifuge tubes. The solution was then centrifuged at high speed (3000 rpm) for 5 min and clear supernatant was obtained. The amount of dexibuprofen released was then measured on UV spectrophotometer (Shimadzu, NAKAGYO-KU, Kyoto 604-8511, Japan) after hydrolysis in buffer solutions at 230–215 and 280–260 nm respectively.

### 3.6. Pharmacology

#### Animals

The animals used for this study were 20–30 g weight mice either male or female and were housed at animal house department of Pharmacology, University of Sargodha. Animals were provided standard diet and water at libitum. All animals were treated according to instructions of National Institute of Health (NIH) guidelines. The study protocol was approved by the ethical Committee, Faculty of Pharmacy, University of Sargodha in the month of June 2014 vide project approval number PHRM-UOS-125.

### 3.7. Animal Care

#### 3.7.1. Temperature and Humidity

The temperature range of animal house was 26–34 °C and relative humidity was kept in a controlled range of 30% to 70%.

#### 3.7.2. Ventilation and Air Quality

The proper ventilation was provided to animals with quality air and stable environment. Animals were avoided from direct exposure to high velocity air (drafts) during the experiment. Heating, ventilation, and air conditioning (HVAC) systems were used to maintain the ventilation rates in accordance with heat load and other variables. Ten to fifteen fresh air changes per hour in animal house were acquired to maintain macro-environmental air quality by constant volume systems and may also ensure micro-environmental air quality.

#### 3.7.3. Drug Administration

The solutions/suspensions of drugs were administered to the animals by either oral or subcutaneous routes. For oral administration oral gavage tube was used while for subcutaneous administration syringe was used.

### 3.8. Anti-Inflammatory Activity

#### Carrageenan Induced Paw Edema in Mice

Anti-inflammatory activity of mutual prodrugs **5a**–**c** was determined by following the already reported method by slight modification against carrageenan induced paw edema in mice [42]. The animals were distributed into 5 groups each having five mice. The mice used for the experiment were kept on fasting for 24 h before the starting of experiment. The animals in Group I were treated with normal saline 10 mL/kg p.o. (per oral) served as control group and Group II was treated with standard dexibuprofen 20 mg/kg p.o. The animals of Groups III, IV and V were treated with prodrugs **5a**–**c**, respectively, as 20 mg/kg p.o. The inflammation was induced after 1 h administration of prodrugs by injecting 0.1 mL freshly prepared carrageenan suspension (1%) in normal saline into the sub planar surface of the right hind paw. The paw volume was measured at 0, 1, 2, 3 and 4 h after the administration of carrageenan by using vernier caliper.

(1) Percent inhibition (%) = 100 (1−X/Y)

where *X* = mean increase in paw thickness of treated mice; and *Y* = mean increase in paw thickness of control mice.

### 3.9. Albumin Induced Inflammation in Mice

Anti-inflammatory potential of synthesized prodrugs **5a**–**c** against egg albumin induced inflammation was also determined for further verification by following the previously reported method [43]. The animals were divided into five groups, each group having five animals. The animals were kept on fasting for 24 h before the beginning of the experiment. The mice in control group (Group I) were treated with normal saline 10 mL/kg p.o. and animals of Group II were treated with dexibuprofen 20 mg/kg p.o. The synthesized prodrugs **5a**–**c** were given to Groups III–V, respectively, as 20 mg/kg p.o. After 1 h of treatment, 0.1 mL of fresh egg albumin was injected into the paw for the induction of inflammation and the paw volume was calculated at 0, 1, 2 and 3 h after the administration of inflammatory agent.

### 3.10. Analgesic Activity

#### 3.10.1. Acetic Acid Induced Writhing in Mice

Koster et al., reported method was adopted for the determination of analgesic activity of the mutual prodrugs **5a**–**c** [44]. The mice were divided in to five groups each group consists of five animals. The control group (Group I) was treated with 10 mL/kg normal saline p.o. while Group II was treated with dexibuprofen 20 mg/kg p.o. The synthesized mutual prodrugs **5a**–**c** (20 mg/kg p.o.) were given to Groups III–V, respectively. For induction of writhings, acetic acid solution (0.6% in normal saline) was given to every mouse post 30 min of treatment. After 5 min of injection of this solution, the number of writhings was counted up to 15 min. The analgesic activity of synthesized mutual prodrugs **5a**–**c** and dexibuprofen was determined by comparing the results with those of the animals in the control group.

#### 3.10.2. Formalin Induced Licking in Mice

Formalin induced licking in mice was also used to determine analgesic activity of prodrugs **5a**–**c** for further verification [45]. The animals were distributed in to five groups having five mice in each group. Group I served as control group was treated with 10 mL/kg normal saline p.o. while Group II was treated with dexibuprofen 20 mg/kg p.o. The prodrugs **5a**–**c** (20 mg/kg p.o.) were administered to Groups III–V, respectively. Formalin (0.05 mL, 2.5%) was injected into sub-planter of right hind paw and responses were observed for 30 min.

### 3.11. Antipyretic Activity

Antipyretic activity of prodrugs **5a**–**c** was evaluated against yeast induced pyrexia in mice according to Sakande et al., reported method [46]. Aqueous suspension of brewer′s yeast (20% in distilled water) 10 mg/kg was given subcutaneous to mice. The rectal temperatures of mice were noted after 16 h of administration of brewer′s yeast. Only, those animals were further used in which the pyrexia had been induced. The animals were divided in to five groups having five mice in each group. The Group I (control group) was treated with 10 mL/kg normal saline p.o. while Group II (Standard Group) was treated with dexibuprofen 20 mg/kg p.o. The prodrugs **5a**–**c** (20 mg/kg p.o.) were administered to Groups III–V, respectively. Then animals were treated as described earlier with control, standard and prodrugs **5a**–**c**. Rectal temperature of animals was recorded at 1, 2, 3 and 4 h after drug administration.

### 3.12. Ulcerogenic Studies

The ulcerogenic potential of dexibuprofen and synthesized mutual prodrugs **5a**–**c** were determined by following the fasted rat model. The animals were categorized into 6 groups, each has 5 animals. Group 1 was control and given 0.5% carboxymethyl cellulose (CMC) suspension. The standard dexibuprofen at a dose of 20 mg/kg p.o. was given to group II. The prodrugs **5a**–**c** (20 mg/kg p.o.) were given to Groups III–V, respectively. Food and water was removed from animal cages 24 h before starting the experiments. After 4 h administration of dose, the animal′s stomach was removed by sacrificing the animal. Stomach was kept on saline soaked filter paper for inspection. The stomach was opened along larger curvature and cleaned with distilled water. The mucous was removed and the area of ulcers and stomachs were noted [47,48].

Following formula was used for calculation of Ulcer index (*UI*) [49];

(2) $$UI=\frac{\mathrm{Area}\;\mathrm{of}\;\mathrm{Ulcer}}{\mathrm{Area}\;\mathrm{of}\;\mathrm{Stomach}}\times 100$$

### 3.13. Ex Vivo Antiplatelet Aggregation Activity

The antiplatelet activity of synthesized mutual prodrugs **5a**–**c** was investigated by following the already reported method with some modifications [50]. Male Sprague-Dawley (SD) rats weighing 320–350 g were used after overnight fasting. Rats were orally administered via gastric tube 100 mg/kg of prodrugs solution or 50 mg/kg of acetyl salicylic acid (ASA) suspended in 0.5% carboxymethyl cellulose (CMC) solution. Blood was collected 90 min after sample administration and platelet-rich plasma (PRP) was prepared by centrifugation of blood at 120× *g* for 15 min and further centrifuged at 850× *g* for 15 min to prepare platelet-poor plasma (PPP). Platelet aggregation was induced by 32.8 µg/mL of collagen or 1.3 µmol/L of adenosine diphosphate (ADP) in 300 µL of PRP. The PRP (300 µL) was incubated at 37 °C for 2 min in the aggregometer with stirring at 1000 rpm and then stimulated with ADP, collagen, epinephrine and A23187. The changes in light transmission were recorded for 5 min after stimulation. The synthesized mutual prodrugs **5a**–**c** were added to the platelet suspension 3 min before the addition of the aggregating agents. The extent of inhibition of platelet aggregation is expressed as percent inhibition (*X*) using the following formula:

(3) X=((A−B)/A)×100

where *A* = maximal aggregation of the control; and *B* = maximal aggregation of sample-treated PRP.

### 3.14. Statistical Analysis

The results are expressed as means ± SEM and Parametric data were compared to control group. One-way ANOVA followed by Dunnet′s test and two way ANOVA followed by Bonferroni *post*-test were applied to assess the data. Values of *p* < 0.05 were considered as statistically significant.

### 3.15. Protein Structure from PDB

The three dimensional crystal structure of mice cyclooxygenase-2 (COX-2) was retrieved from the Protein Data Bank (PDB) with PDBID 3NTG. The overall stereo-chemical properties of cyclooxygenase-2 structure and Ramachandran graph and values [51] were assessed by Molprobity server [52].

### 3.16. Prodrugs Preparation

The parent dexibuprofen and prodrugs **5a**–**c** were sketched in ACD/ChemSketch tool. The designed structures were further visualized and minimized by UCSF Chimera 1.10.1 [53]. The Molinspiration (Available online: http://www.molinspiration.com/) and Molsoft (Available online: http://www.molsoft.com/) online computational tools were applied to predict the drug-likeness and basic biological properties of synthesized prodrugs. Moreover, Lipinski′s rule of five was analyzed using Molsoft and Molinspiraion tools. The number of rotatable bonds, H-bond acceptors (HBA) and H-bond donors (HBD) were also confirmed by PubChem (Available online: https://pubchem.ncbi.nlm.nih.gov/).

### 3.17. Grid Generation and Molecular Docking

The structure of target enzyme COX-2 was prepared and optimized to perform molecular docking studies. Hydrogen atoms were added to the target enzyme and bond order was also assigned. Molecular docking was carried out and different docked complexes were visualized by PyRx tool (vina wizard) [54]. The grid box parametric central values of *X* = 28.6432, *Y* = 28.6975 and *Z* = 9.4725, while, size of *X* = 59.75, *Y* = 77.94 and *Z* = 63.86, respectively, with spacing 1.0 Å and exhaustiveness values were adjusted to attain the finest binding conformational poses of ligand molecules. The maximum docking poses (100 runs) for each docking were adjusted to get the best docking complex with good conformational pose. All the synthesized mutual prodrugs **5a**–**c** and dexibuprofen were docked separately against crystal structure of COX-2 and the obtained docked complexes were further evaluated on lowest binding energy (kcal/mol) values. Docking analysis was employed by Discovery Studio (2.1.0) [55] and UCSF Chimera 1.10.1.

### 3.18. Molecular Dynamics (MD) Simulation

The best docked energy (Kcal/mol) complexes were selected for molecular dynamics (MD) simulation through Groningen Machine for Chemicals Simulations (GROMACS) 4.5.4 package [56] with GROMOS 53A6 force field and water model SPC216 [57]. The topology files (receptor and ligand) were produced by using GROMOS 53A6 force-field and online PRODRG Server [58], respectively. Furthermore, all the receptor-ligand complexes were solvated and placed in the center of cubic box with an adjusted minimum 0.9 Å distance. To neutralize the system charge ions were added before the energy minimization. Energy minimization (nsteps = 50,000) was done by steepest descent method (1000 ps) and energy calculation was done by Particle Mesh Ewald (PME) method [59], while for covalent bond constraints linear constraint solver (LINCS) algorithm was used [60]. The NVT analysis was also applied for 100 ps at a fixed volume, pressure (1atm) and temperature (300 K) to equilibrate the system with protein and ligands [61]. The final MD run was set to 5000 ps with nsteps 2,500,000 for each protein-ligand complexes and trajectories files analysis was done using Xmgrace (Available online: http://plasma-gate.weizmann.ac.il/Grace/). 

## 4. Conclusions

The present work utilizes safer promoieties, i.e., natural antioxidants, for the synthesis of dexibuprofen prodrugs **5a**–**c**. The spectroscopic data of these prodrugs confirmed their structures. All synthesized mutual prodrugs showed encouraging hydrolysis rate in SIF + 80% human plasma probably due to the presence of esterase enzymes in plasma. The synthesized prodrugs are stable in gastric mucosa at pH 1.2 and are not hydrolyzed to free dexibuprofen. On the other hand, in simulated intestinal fluid pH 7.4, 18% of the prodrugs are hydrolyzed to free drug. The prodrugs synthesis also prevents the accumulation of drug in gastric mucosa, resultingin decreased GI irritation without the loss of pharmacological response. The molecular docking and simulation studies also proved that prodrugs formed stable complexes with the Cox-2 protein, thus supporting our wet lab results. The ulcerogenic activity and ex vivo antiplatelet aggregation activity results confirmed that synthesized prodrugs are less irritant to gastrointestinal mucosa than dexibuprofen. It is concluded, therefore, that the synthesized prodrugs have promising pharmacological activities with reduced gastrointestinal adverse effects compared to the parent drug.

## Figures and Tables

**Table 1:** Effect of mutual prodrugs (**5a**–**c**) in carrageenan induced edema (mm) in mice.

Serial. No.	Inflammation at 0 h (mm)	Inflammation at 1 h (mm)	Inflammation at 2 h (mm)	Inflammation at 3 h (mm)	Inflammation at 4 h (mm)
Dose	Mean ± SEM	Mean ± SEM	Mean ± SEM	Mean ± SEM	Mean ± SEM
Control 10 mg/kg	4.17 ± 0.08	4.40 ± 0.10	4.6 ± 0.06	4.85 ± 0.06	5.07 ± 0.06
Dexibuprofen 20 mg/kg	4.37 ± 0.06	4.35 ± 0.06 ^ns^ (1.1%)	4.07 ± 0.02 *** (11.71%)	3.90 ± 0.04 *** (19.58%)	3.75 ± 0.06 *** (26.03%)
**5a** 20 mg/kg	4.42 ± 0.05	4.50 ± 0.04 ^ns^ (−2.27%)	4.25 ± 0.06 ** (7.80%)	4.12 ± 0.06 *** (15.05%)	3.92 ± 0.02 *** (22.68%)
**5b** 20 mg/kg	4.37 ± 0.04	4.22 ± 0.1 ^ns^ (4.09%)	4.25 ± 0.06 ** (7.80%)	4.15 ± 0.02 *** (14.43%)	3.92 ± 0.05 *** (22.68%)
**5c** 20 mg/kg	4.32 ± 0.04	4.22 ± 0.04 ^ns^ (4.09%)	4.05 ± 0.02 *** (12.14%)	3.80 ± 0.04 *** (21.64%)	3.60 ± 0.04 *** (28.99%)

Results are expressed as means ± SEM. Significant at, ** = *p* < 0.01, *** = *p* < 0.001, ^ns^ = non-significant when compared to control. Values in bracket indicate percent inhibition.

**Table 2:** Effect of mutual prodrugs (**5a**–**c**) in egg albumin induced paw edema (mm) in mice.

Treatment/Dose	Inflammation at 0 h (mm)	Inflammation at 1 h (mm)	Inflammation at 2 h (mm)	Inflammation at 3 h (mm)
Dose	Mean ± SEM	Mean ± SEM	Mean ± SEM	Mean ± SEM
Control 10 mL/kg	4.35 ± 0.05	4.40 ± 0.10	4.80 ± 0.10	5.22 ± 0.07
Dexibuprofen 20 mg/kg	4.05 ± 0.41	3.20 ± 0.04 *** (27.27%)	3.42 ± 0.20 *** (28.75%)	3.00 ± 0.00 *** (42.52%)
**5a** 20 mg/kg	4.67 ± 0.08	3.70 ± 0.16 * (15.90%)	3.37 ± 0.20 *** (29.79%)	3.30 ± 0.04 *** (36.78%)
**5b** 20 mg/kg	4.15 ± 0.32	3.95 ± 0.02 ^ns^ (10.22%)	3.40 ± 0.20 *** (29.16%)	3.17 ± 0.11 *** (39.27%)
**5c** 20 mg/kg	4.60 ± 0.33	3.12 ± 0.06 *** (29.09%)	3.02 ± 0.06 *** (37.08%)	2.92 ± 0.07 *** (44.06%)

Results are expressed as means ± SEM. Significant at * = *p* < 0.01, *** = *p* < 0.001, ^ns^ = non-significant when compared to control. Values in bracket indicate percent inhibition.

**Table 3:** Effect of prodrugs (**5a**–**c**) against acetic acid induced writhing and formalin induced licking in mice.

Treatment/Dose	No. of Writhing	Lickings Time
Dose	Mean ± SEM	Mean ± SEM
Control 10 mL/kg	37.20 ± 1.10	2.83 ± 0.26
Dexibuprofen 20 mg/kg	17.06 ± 1.08 *** (54.12%)	1.60 ± 0.22 *** (43.46%)
**5a** 20 mg/kg	15.00 ± 1.29 *** (59.67%)	0.70 ± 0.22 *** (75.26%)
**5b** 20 mg/kg	17.50 ± 0.64 *** (52.95%)	0.98 ± 0.17 *** (65.37%)
**5c** 20 mg/kg	18.25 ± 0.85 *** (50.94%)	1.31 ± 0.07 *** (53.71%)

Results are expressed as means ± SEM. Significant at *** = *p* < 0.001 when compared to control. The figure in brackets represents percentage inhibition.

**Table 4:** Effect of prodrugs (**5a**–**c**) against yeast induced pyrexia (°F) in mice.

Treatment/Dose	Temperature before Induction (°F)	After 24 h of Induction (°F)	1 h (°F)	2 h (°F)	3 h (°F)
Dose	Mean ± SEM	Mean ± SEM	Mean ± SEM	Mean ± SEM	Mean ± SEM
Control (10 mL/kg)	95.17 ± 0.34	96.02 ± 0.44	96.25 ± 0.39	96.22 ± 0.39	96.22 ± 0.39
Dexibuprofen (20 mg/kg)	95.20 ± 0.21	95.77 ± 0.26	95.25 ± 0.37 ^ns^	95.02 ± 0.35 *	94.97 ± 0.31 *
**5a** (20 mg/kg)	94.37 ± 0.04	95.14 ± 0.02	94.97 ± 0.06 *	94.62 ± 0.08 **	94.37 ± 0.08 ***
**5b** (20 mg/kg)	94.25 ± 0.10	95.47 ± 0.19	94.87 ± 0.06 **	94.5 ± 0.12 ***	94.17 ± 0.06 ***
**5c** (20 mg/kg)	98.95 ± 0.04	102.1 ± 0.96	98.35 ± 0.05 ***	97.65 ± 0.15 **	97.25 ± 0.08 ^ns^

Results are expressed as means ± SEM. Significant at * = *p* < 0.05, ** = *p* < 0.01, *** = *p* < 0.001, ^ns^ = non-significant when compared to control.

**Table 5:** Ulcerogenic activity of synthesized mutual prodrugs (**5a**–**c**) and Dexibuprofen.

Serial. No.	Compounds	Ulcer Index (Mean±SEM)
Control group	CMC *	0.37 ± 0.37
1	Dexibuprofen	2.89 ± 0.63
2	**5a**	1.55 ± 0.09 **
3	**5b**	1.34 ± 0.06 **
4	**5c**	1.61 ± 0.58 *

CMC * = Carboxymethyl cellulose, Values are expressed as Mean ± SEM, (*n* = 6) by one way ANOVA (followed by Tukey) test, Significant at * = *p* < 0.05, ** = *p* < 0.01 vs. Dexibuprofen.

**Table 6:** Docking results of pro-drug compounds using AutoDockVina.

Prodrugs	Docking Energy (kcal/mol)	Binding Pocket Residues
**5a**	−8.90	Gln189, His193, Lys197, Leu377, Phe390, Val281, Leu280, val273, Glu276, Gln275, His
**5b**	−9.90	Gln189, His193, Thr192, Ala188, Phe186, Tyr371, Trp373, His374, Leu276, Leu377, Ala429, Val430,Val433, Phe390, Leu394, Val281, Leu280
**5c**	−9.40	His193, Thr192, Ala188, Phe185, Val277, Lys197, Thr198, His372, Tyr371, His374, Leu377, Ala185
Dexibuprofen	−8.60	Gln189, Phe390, Val281, Leu263, Leu394 and Leu377

## Supplementary Materials

Supplementary materials can be found at www.mdpi.com/1422-0067/17/12/2151/s1.
