# Supplementary material for: Synthesis, Bioevaluation and Molecular Dynamic Simulation Studies of Dexibuprofen–Antioxidant Mutual Prodrugs
Source: Int J Mol Sci. 2016 Dec 21;17(12):2151. doi: 10.3390/ijms17122151 (PMC5187951; doi:10.3390/ijms17122151)
Supplement: Supplementary file 1 [file ijms-17-02151-s001.pdf]

Supplementary Materials: Synthesis, Bioevaluation and Molecular Dynamic Simulation Studies of Dexibuprofen–Antioxidant Mutual Prodrugs

Zaman Ashraf, Alamgeer, Raqiqatur Rasool, Mubashir Hassan, Haseeb Ahsan, Samina Afzal, Khurram Afzal, Hongsik Cho and Song Ja Kim

Table S1. Chemo-informatics and biological properties of pro-drugs.

Properties	5a	5b	5c
Molecular formula	$C_{25}H_{38}O_4$	$C_{22}H_{24}O_6$	$C_{24}H_{24}O_6$
Mol. wt. (g/mol)	402.28	384.16	408.16
No. of HBA	4	6	6
No. of HBD	0	0	0
Log*P*	6.40	4.77	4.48
Polar surface area (PSA) (Å^2)	40.80	58.27	61.56
Molar refractivity (cm^3)	116.26	102.69	110.04
Density (g/cm^3)	1.03	1.20	1.21
Surface tension (dyne/cm)	38.3	46.4	48.1
Polarizability (cm^3)	46.08	40.71	43.62
Molecular volume (Å^3)	434.97	384.82	420.59
Drug score	0.83	1.22	1.04
Lipinski rule validation	Yes	Yes	Yes

Table S2. The binding energy values of all docked complexes.

Docking Poses	Ligand Binding	rmsd/ub	rmsd/lb
**5a.1**	−8.9	0	0
**5a.2**	−8.1	3.286	2.031
**5a.3**	−7.9	10.507	3.82
**5a.4**	−7.7	5.816	3.182
**5a.5**	−7.6	39.83	36.351
**5a.6**	−7.5	33.837	30.689
**5a.7**	−7.5	33.489	30.54
**5a.8**	−7.4	17.198	12.403
**5a.9**	−7.2	7.212	4.224
**5b.1**	−9.9	0	0
**5b.2**	−9.4	2.62	1.45
**5b.3**	−8.8	10.171	3.072
**5b.4**	−8.8	33.575	31.883
**5b.5**	−8.6	10.531	3.546
**5b.6**	−8.5	29.318	27.418
**5b.7**	−8.4	29.293	27.533
**5b.8**	−8.4	8.035	4.775
**5b.9**	−8.2	32.024	30.507
**5c.1**	−9.4	0	0
**5c.2**	−9.2	4.899	3.577
**5c.3**	−9.1	6.712	4.028
**5c.4**	−8.8	5.662	4.427
**5c.5**	−8.6	32.869	29.096
**5c.6**	−8.6	29.864	27.537
**5c.7**	−8.6	9.727	3.118
**5c.8**	−8.5	2.278	1.445
**5c.9**	−8.5	9.933	2.601

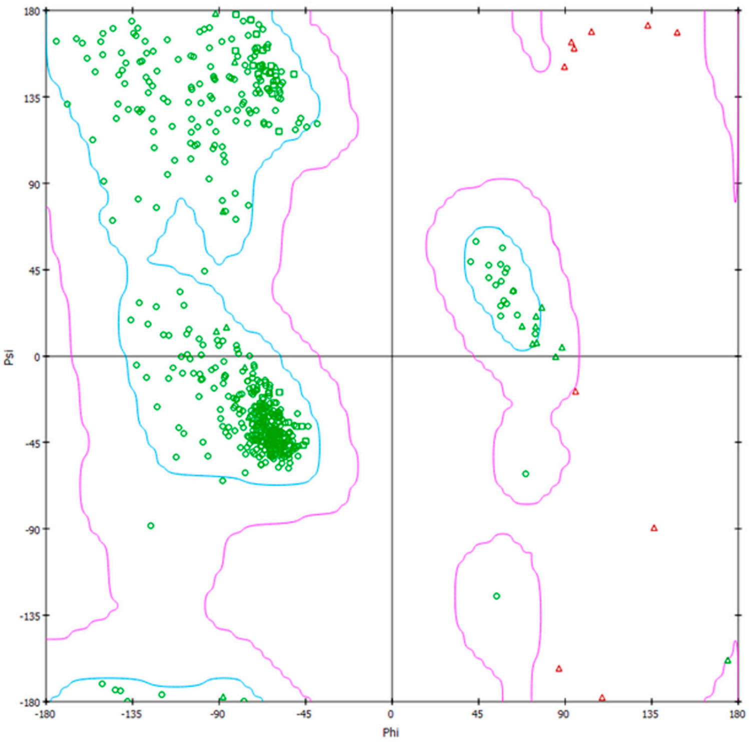

Figure S1. Ramachandran graph of target protein cycooxygenase-2, green (residues in favourable region), red (residues in allowed region).
